# Thyroid dysfunction during pregnancy

**DOI:** 10.1186/1756-6614-8-S1-A15

**Published:** 2015-06-22

**Authors:** Malgorzata Karbownik-Lewinska

**Affiliations:** 1 Department of Oncological Endocrinology, Medical University of Lodz, 7/9 Zeligowski St., 90-752 Lodz, Poland; 2 Department of Endocrinology and Metabolic Diseases, Polish Mother’s Memorial Hospital – Research Institute, Lodz, Poland

## 

Pregnancy is characterized by specific changes in thyroid physiology. First, the requirements for dietary iodine increase substantially, which is due to the increased thyroid hormone formation, the enhanced iodine metabolism, the increased loss of this nutrient, etc. According to the current recommendations, the supply of iodine during gestation, and also during lactation, should be at least 250 micrograms daily. Therefore, additional iodine supplementation is advised at the level of at least 150 micrograms daily to be administered to every pregnant and lactating woman. According to current recommendations, this additional iodine supplementation is also recommended in women who are going to be pregnant. Thyroid hyperstimulation, caused by human chorionic gonadotrophin (hCG) at the end of the first trimester, is another physiological change during pregnancy. Concerning thyroid disorders in pregnant women, thyroid dysfunctions, i.e. hypo- and hyperthyroidism, are most frequent. The diagnosis is based on abnormal values of thyroid hormones and thyrotropin concentrations, although reference ranges differ substantially from those accepted for general population. Thus, the interpretation of obtained results are difficult. According to the current recommendations, the estimated upper limit of TSH concentration during preconception and pregnancy is 2.5 mIU/L, however still some difficulties in the interpretation of results occur mainly in the first trimester. In turn, the lack of reference ranges for thyroid hormones is associated with severe diagnostic problems especially in the 2^nd^ and 3^rd^ trimesters. Thyroid antibodies should be screened before and/or during pregnancy and possibly monitored in subjects with thyroid dysfunction, especially with hyperthyrodism. Both thyroid dysfunctions are predominantly of autoimmune etiology, with hypothyroidism occurring much more frequently. The prevalence of hypothyroidism, especially of its subclinical form during preconception and gestational state, is estimated at the level of 10-15% or possibly higher.

The etiology of hypothyroidism is usually associated, as in the general population, with Hashimoto’s thyroiditis. Replacement therapy with levothyroxine (L-thyroxine) is the treatment of choice in hypothyroidism. Hypothyroid patients on L-thyroxine replacement should be carefully monitored to keep TSH and thyroid hormone concentrations in recommended ranges before conception and during pregnancy. Patients with pre-existing hypothyroidism generally require increased L-thyroxine doses during pregnancy. In turn, hyperthyroidism in pregnancy is usually associated with Graves’ disease. Medical treatment in hyperthyroid pregnant women is the management of choice, with propylthiouracil being the preferred antithyroid drug in the first trimester and thiamazole being recommended in the 2^nd^ and 3^rd^ trimesters. Careful control of maternal thyroid function is required during antithyroid drug treatment to avoid fetal hypothyroidism. The increased concentration of hCG in the first trimester assumes relatively frequently the form of gestational transient thyrotoxicosis. This form of thyrotoxicosis constitutes the separate entity; it usually needs no treatment, although in some patients with severe clinical symptoms the treatment with antithyroid drugs may be useful. Summing up, the diagnostic and treatment procedures in pregnant women with thyroid dysfunction are characterized by certain specificity and should be updated due to results of ongoing epidemiological studies, especially those on establishment of reference ranges of thyroid hormones.

